# When Less Is Good, Is None Better? The Prognostic and Therapeutic Significance of Peri-Transplant Minimal Residual Disease Assessment in Pediatric Acute Lymphoblastic Leukemia

**DOI:** 10.3390/jcm6070066

**Published:** 2017-07-07

**Authors:** Adam Lamble, Rachel Phelan, Michael Burke

**Affiliations:** 1 Pediatric Hematology/Oncology, Oregon Health & Science University, Portland, OR 97239, USA; lamble@ohsu.edu; 2 Pediatric Hematology/Oncology/Blood and Marrow Transplant, Medical College of Wisconsin, Milwaukee, WI 53226, USA; rphelan@mcw.edu

**Keywords:** ALL, pediatric, transplant, leukemia, minimal residual disease

## Abstract

The measurement of minimal residual disease (MRD) in pediatric acute lymphoblastic leukemia (ALL) has become the most important prognostic tool of, and the backbone to, upfront risk stratification. While MRD assessment is the standard of care for assessing response and predicting outcomes for pediatric patients with ALL receiving chemotherapy, its use in allogeneic hematopoietic stem cell transplant (HSCT) has been less clearly defined. Herein, we discuss the importance of MRD assessment during the peri-HSCT period and its role in prognostication and management.

## 1. Introduction

Cure rates for pediatric acute lymphoblastic leukemia (ALL) are currently >90%, in large part due to steady improvement though large cooperative group trials [1]. Despite these improvements, there remains a subset of high-risk (HR) patients for which outcomes remain poor. For these patients, allogeneic hematopoietic stem cell transplantation (HSCT) can be used as consolidative therapy in the upfront or relapse setting, to improve outcomes. Great strides have been made in HSCT over the last several decades to further improve these outcomes, including improvements in donor registries, use of high resolution human leukocyte antigen (HLA) typing, and better supportive care [2]. However, despite these advances, short and long term complications continue with post-HSCT and remission is often not sustained. In fact, death from relapsed leukemia remains the most common event (post-HSCT) for hematologic malignancies [3,4]. 

At its most basic level, the way we identify which patients may benefit most from HSCT is when their risk of mortality with conventional chemotherapy becomes greater than their risk of mortality following HSCT. This decision must take into account the risk of relapse and potential complications associated with both therapies. The ability to identify HR patients who would most benefit from HSCT is constantly evolving. Current evidence supports HSCT in patients who experience primary induction failure (PIF), have an early first relapse (defined as <36 months from initial diagnosis of ALL), or any second or greater relapse [5,6]. While no absolute indications exist, there are also specific genetic subtypes that may benefit from HSCT in first remission (CR1). Previously, this group included patients with severe hypodiploidy (<44 chromosomes and/or DNA index < 0.81) and those with Philadelphia-chromosome positive ALL (Ph+ ALL) [7]. However, recent evidence with tyrosine kinase inhibitors (TKIs), in combination with conventional chemotherapy, challenges the Ph+ ALL indication of HSCT in CR1 [8,9], and patients with severe hypodiploidy (who achieve MRD negativity at end of Induction) might also not need HSCT to improve their predicted event-free survival (EFS) [10]. However, minimal residual disease (MRD) at the end of induction (EOI) and/or consolidation (EOC) has emerged as a possible indication for HSCT in CR1, particularly in patients with HR B-ALL who remain MRD-positive at EOC (where EFS is <40% [11,12,13]). Just as the indication for HSCT in these two genetic subtypes has evolved, we can anticipate that by expanding the role of MRD to the peri-transplant setting, we may be able to better define the group of patients who would benefit most from HSCT rather than from chemotherapy alone. This review will highlight the current methodologies available for MRD assessment in pediatric ALL, its role in risk stratification, and how peri-transplant MRD remains prognostic and might be managed to improve pediatric outcomes.

## 2. MRD Assays in Pediatric ALL

ALL has long been the prototypical disease utilizing MRD assessment for risk stratification, and remains an ideal candidate for emerging MRD techniques (Table 1) [14]. This is primarily related to the presence of both an aberrant immunophenotype and/or specific genetic aberrations, including gene rearrangements in the immunoglobulin (IG) or T cell receptor (TCR) gene for B-cell ALL and T-cell ALL, respectively. The advantages and disadvantages for different MRD assays currently used in pediatric ALL are summarized below.

### 2.1. Multicolor Flow Cytometry

The sequential expression of specific antigens during the normal maturational process of immature lymphocytes has been previously well described [15]. Leukemia cells may under express, overexpress, and/or inappropriately express lineage-specific antigens, leading to an aberrant immunophenotype. In the case of precursor B-cell ALL, the over expression of CD10 and/or the under expression of CD45RA is present in over 80% of cases [16]. The concept of utilizing this aberrant immunophenotype as not only a way to identify leukemia cells, but also a way of quantifying MRD, first emerged through the unitization of immunofluorescence [14,17]. This concept was further expanded with the advent of 4-color flow cytometers in the 1990s. Since then, most diagnostic laboratories have expanded to 8–10-color flow cytometers. Multicolor Flow Cytometry (MFC) doesn’t quite reach the sensitivity of the polymerase chain reaction (PCR)-based assays (<10^−4^ versus <10^−5^, respectively), but studies comparing the two modalities show analogous results [18,19,20,21]. Of note, the EuroFlow consortium has recently introduced newer techniques designed to achieve an even greater sensitivity detection rate (<10^−5^) for MFC, through the addition of more flow channels, combined with a multivariate analysis, which will allow for a better delineation between the hematogone and leukemia populations [22]. Strengths of traditional MFC for MRD include the lower cost of analysis, the rapidity of turnaround for results, and the lack of a necessary diagnostic sample to perform the assay. Beyond the sensitivity limits, other limitations to MFC include potential variations between laboratories and the difficulty distinguishing hematogones (earliest of B-cell precursors found in the bone marrow) from leukemia blasts. Overall, MFC has been widely adopted in the United States and is considered the standard of care for detecting MRD in pediatric ALL.

### 2.2. Molecular/Allele-Specific Oligonucleotide PCR-Based

Southern blotting set the precedent for using clonal IG and TCR genes found in ALL as a means of disease detection. This technique relies on the different combinations of the rearranged variable, diversity, and joining (V-D-J) genes. While the sensitivity of this approach has limited its clinical utility, it laid the foundation for future, more optimized techniques for MRD detection such as PCR [23]. Invented in the late 1980s and further developed in the 1990s with the addition of real-time quantitation, PCR is a fast and cheaper way to amplify a single copy of DNA for disease monitoring. Building on the concepts established by southern blotting, but focusing on the highly diverse junctional regions (instead of sequencing the entire V-D-J region), PCR provides a higher sensitivity, sufficient for MRD detection [14]. Alternatively, ALL with specific chromosomal rearrangements, such as BCR-ABL (harboring chromosome 9 and 22 translocation), and ETV6-RUNX1 (harboring chromosome 12 and 21 translocation), can be analyzed using reverse transcriptase PCR (RT-PCR). However, this approach has been limited to less than half of ALL diagnoses due to their mutational frequencies [24,25]. Strengths for PCR-based MRD over MFC primarily include a higher sensitivity, but this may be outweighed by the cost of the assay, the need of a diagnostic sample to generate leukemia-specific primers, the need for assay expertise, and having a longer lag time to get results. In addition, there can also be false positives due to nonspecific binding of the primer during hematogone regeneration. Despite the limitation of PCR-based MRD assessment, it has been widely adopted in Europe and has become the standard of care for MRD assessment in their clinical trials for pediatric ALL.

### 2.3. High Throughput Sequencing PCR

The development of high-throughput sequencing PCR (HTS-PCR) has been the newest technique to enter the field of MRD detection. Defined by its ability to sequence millions of sequences in parallel, HTS-PCR allows for deep sequencing of the entire V-D-J domain of the IG and TCR gene, similar to the early southern blotting techniques. This results in amplification of all potential rearrangements in a sample and allows for a higher sensitivity, due to a more comprehensive analysis. In addition, it becomes possible to detect clonal evolution of rearrangements which are known to occur in some patients. 

Results using this technique for MRD evaluation were first published by a group studying chronic lymphocytic leukemia (CLL) [26]. Shortly following this study, the use of HTS-PCR was reported by two groups for the assessment of MRD in adult ALL [27,28]. The benefit of HTS-PCR for MRD was further highlighted retrospectively in 98 ALL samples collected through the Children’s Oncology Group (COG) [29]. Ninety-one of the 98 patients (93%) had an IG that was sufficient for study. HTS-PCR was able to detect MRD in all of the cases that were positive by flow cytometry and with no false positives identified. In addition, HTS-PCR identified 28 patients as positive MRD that had no MRD detected by conventional MFC. The majority of the non-overlap can likely be attributed to the greater sensitivity of HTS-PCR, which further strengthens its potential to improve clinical prognostication in ALL. In addition, 5 samples were identified by HTS-PCR with MRD > 10^−4^ that were negative using MFC. Theoretically, these samples should have been detected by conventional MFC MRD, given the sensitivity of this technique, but were missed. The authors hypothesized that this could have been the result of therapy-induced immunophenotypic maturation missed by MFC that led to the false negatives. This concept was further supported by triple flow sorting for mature B-cells in 10 patient samples that had negative MRD by MFC at the end of induction therapy. In one of these samples, they identified the same clonal V-D-J rearrangement in B-cells that not only lacked the aberrant immunophenotype of the diagnostic malignant clones but had an immunophenotype consistent with a mature B-cell. As this study was a proof of principle, attempting to show feasibility and non-inferiority compared to traditional techniques, they did not assess the non-overlapped patients for clinical outcomes. It is unclear what clinical significance the non-overlapping group represents. MRD levels less than 10^−5^ may be cleared by normal immune surveillance, and may therefore be clinically insignificant. In addition, cells that undergo maturational drift may lose their malignant potential and, similarly, be clinically insignificant. Further studies are needed to assess the prognostic potential of HTS-PCR for the evaluation of MRD, and whether it can eventually supplant the traditional modalities for MRD risk stratification in ALL.

## 3. Prognostic Significance of MRD

### 3.1. Pre-HSCT MRD

The Pre-HSCT period is ideally a time when patients have a low, if not absent, leukemic burden; furthermore, it has long been shown that patients in morphologic CR pre-HSCT have far better outcomes than those not in CR [30,31]. Taken further, those with an unmeasurable disease pre-HSCT may have even better outcomes than those in morphologic CR but with a measurable disease. The benefit of negative MRD in the pre-HSCT setting was first reported in the early 1990s [17,32]. With the evolution of multiple MRD technologies, a number of retrospective studies followed, investigating the prognostic value of pre-HSCT MRD using different MRD techniques and sensitivity cutoffs (Table 2). While the majority of MRD data has come to similar conclusions supporting the prognostic significance of pre-HSCT MRD, several questions remain, including: (1) When is the most appropriate time pre-HSCT to test for MRD? (2) What MRD value is most predictive of outcome? (3) Does the timing and/or MRD cutoff depend on the modality used? (4) When does the risk of toxicities related to chemotherapy strategies to obtain MRD negativity outweigh the potential benefit? And lastly, (5) Do MRD observations for groups in first remission equally apply to those in ≥1st relapse?

One of the largest and most widely cited prospective trials performed, assessing MRD analysis in the peri-transplant setting, was through the Berlin-Frankfurt-Munster (BFM) study group [41]. Using PCR-based MRD and looking at only patients in CR2 or greater, Bader et al. initially separated 91 patients into four groups, based on the pre-HSCT level of MRD (Group 1: undetectable MRD; Group 2: detectable MRD < 10^−4^; Group 3: MRD between >10^−4^ and <10^−3^; Group 4: MRD > 10^−3^). Although there were a relatively small number of patients reported in each group, no difference was observed in the EFS or the cumulative incidence of relapse (CIR) between groups 1 and 2, or between groups 3 and 4. Therefore, the authors combined these groups and showed that patients with low MRD (<10^−4^), compared to those with a high MRD (>10^−4^), had a greater predicted EFS (0.60 vs. 0.27, *p* = 0.004) and lower CIR (0.13 vs. 0.57, *p* < 0.001), concluding that low, but not necessarily undetectable, MRD prior to HSCT predicts against relapse.

A more recent prospective trial performed through the Australian and New Zealand Children’s Haematology/Oncology Group (ANZCHOG) supported the above results but expanded their patient cohort to include those in CR1. Studying 81 patients with ALL, which included 41 in CR1 and 40 in ≥CR2, Sutton et al. looked at PCR-based MRD just before HSCT [50]. Those with a negative MRD (<10^−4^) prior to HSCT had a better 5-year OS, whether they were in CR1 (87% vs. 58%, *p* < 0.05) or ≥CR2 (86% vs. 55%, *p* < 0.05). The authors concluded that any level of detectable MRD pre-HSCT significantly increased the risk of relapse, regardless of whether the patient was in first or greater remission. Additionally, within the MRD-positive group, higher levels of MRD (>1 × 10^−2^), when compared to lower levels (<1 × 10^−2^), were associated with a significantly lower 5-year OS (50% vs. 68%, *p* < 0.005).

Similar to PCR-based MRD, MFC has been utilized successfully to support the prognostic significance of low MRD prior to transplant. St. Jude’s Children’s Research Hospital compared pre-HSCT MRD in 64 patients with ALL to 58 patients with acute myeloid leukemia (AML) that were in ≥CR1 (11 whom were not in remission [43]). For both groups, persistent MRD (≥0.1% for AML and ≥0.01% for ALL) at the time of HSCT was associated with a higher rate of relapse and transplant-related mortality (TRM). Conversely, they reported in the ALL cohort only that for every level of reduction in MRD there was an improvement in survival during the observation period (*p* = 0.002). This not only emphasizes the presence of a graft versus leukemia (GVL) effect in AML but also suggests that a reduction of the leukemia burden prior to HSCT may be most beneficial in ALL.

The concept that there is no safe level of MRD prior to HSCT that will not influence survival was recently highlighted in a study using HTS-PCR. Pulsipher et al. hypothesized that by using HTS-PCR and further improving the sensitivity of MRD detection pre-HSCT, one could improve its prognostic significance [49]. The authors retrospectively assessed 41 patients with ALL in CR1 or CR2 enrolled on the COG HSCT trial ASCT0431, and were able to detect leukemia blast percentages as low as 4.2 × 10^−7^. Using a cutoff of 10^−6^, the authors reported that none of the 22 patients who were MRD-negative relapsed post-HSCT. This was contrasted to the 9 of 19 patients (47%) with positive MRD where relapse occurred. This resulted in a 2-year relapse probability of 0% vs. 53% for patients who were MRD-negative and -positive, respectively (*p* < 0.0001). Direct comparisons of these samples showed that HTS-MRD was more successful at predicting relapse and overall survival than the MFC data previously obtained in the study. In addition, unlike the above study from Leung et al., they did not observe a trend in relapse based on the level of MRD, with relapse occurring frequently even at the lowest level of detection. This data argues the importance of complete MRD negativity, prior to HSCT, to ensure the best chance of survival.

### 3.2. Post-HSCT MRD

Not all patients with negative MRD prior to HSCT, as assessed by current modalities, will remain relapse free. This suggests that there are either leukemia cells beneath the level of detection or, possibly, sub-clones present, with different immunophenotypic profiles leading to false negative MRD assessment. Therefore, the measurement of MRD post-HSCT, especially when assessed via serial measurements, may further strengthen the ability to predict relapse. To date, there have been far fewer trials exploring the role of MRD in the post-HSCT setting. Those that have investigated MRD post-HSCT suggest the longer MRD persists, the more prognostic it becomes in predicting relapse and death (Table 3).

Reporting a similar patient cohort as the BFM study group discussed above, Bader et al. assessed MRD using PCR in the bone marrow on days 30, 60, 90, 180, and 365, following HSCT in 113 pediatric patients with relapsed ALL [53]. They reported that for all time points, the level of MRD was inversely correlated with EFS (*p* = 0.004) and positively correlated with CIR (*p* < 0.01). In addition, they identified a threshold of >10^−4^ MRD to provide justification for pre-emptive therapeutic intervention.

Similarly, Balduzi et al. reported post-HSCT results where MRD was measured up to 5 time points post-HSCT [47]. They found that any patient with positive MRD at any of the 5 time points had a greater risk of relapse. This risk further increased the later in the post-HSCT period when the MRD was identified. Additionally, all patients who had high MRD (>10^−3^) (at any time point post-HSCT) relapsed, despite attempts at preventing relapse. 

The COG ASCT0431 ALL HSCT trial assessed MRD post-HSCT prospectively, using MFC and retrospectively using HTS-PCR at 30 days, 100 days, 8 months, and 12 months post-HSCT [49]. In the 53 patients with samples available for analysis with HTS-PCR, they found that 11 of 15 (73%) patients who were MRD-positive (>10^−6^) at any of the time points relapsed, compared to only 5 of 38 (13%) patients who were MRD negative at every time point (*p* < 0.0001). When they compared results based on HTS-PCR versus MFC MRD, HTS-PCR MRD was superior at predicting relapse at every time point. This difference in the detection rate was further highlighted when MFC MRD was unable to detect a significant difference in relapse between MRD-positive and -negative patients at day 30 post-HSCT, whereas HTS-PCR identified a 67% relapse rate for patients who were MRD-positive at day 30 (compared to a 25% risk in those who were MRD negative (*p* = 0.01)). Additionally, there were 11 patients with at least one positive MRD result post-HSCT using HTS-PCR that were negative at every time point using MFC. Seven of these 11 patients eventually relapsed, suggesting the higher sensitivity of HTS-PCR MRD in predicting relapse for patients who had unidentifiable levels of MRD by MFC. Of note, there were three patients with at least one positive post-HSCT MFC MRD that were negative at every time point, using HTS-PCR MRD. Importantly, none of these patients went on to relapse, which suggests that MFC may lead to false positive reports in cases of very low MRD.

Regardless of the modality used to detect MRD, the fact that not all patients with a positive MRD test in the post-HSCT setting will go on to relapse is significant, and could be related to a number of factors. From a technical standpoint, for MFC-based MRD, these false positive cases may represent immunophenotypic overlap between ALL blasts and normal hematogones. As well, MRD detection during early time points post-HSCT may occur prior to any GVL effect. This GVL effect may be responsible for eliminating residual disease later (post-HSCT) and preventing relapse in a subset of patients who have MRD identified early but are able to clear it by a later time point. This is supported by studies that have shown a worse prognosis the later MRD is detected in the post-HSCT setting [47,53]. Similarly, not all patients with persistently negative MRD will remain disease free. This may be related to the frequency and timing of sampling, the rapidity of disease growth, and/or the poor-quality sampling from hypoplastic bone marrows. 

## 4. Therapeutic Implications Based on Peri-HSCT MRD

### 4.1. Pre-HSCT MRD to Minimize Therapy

HSCT is curative for a large subset of patients but is not without its risk of both short and long term complications. While much progress has been made in supportive care to improve outcomes from short-term complications following transplant, the incidence of late effects remains a major concern. Late effects in children following HSCT include central nervous system impairment, decreased linear growth, cardiotoxicity, pulmonary toxicity, infertility, and an increased incidence of secondary cancers [54,55,56,57,58,59]. Patients who go into transplant in second or greater relapse are at risk for even further complications, given the extensive pretreatment they have received. While some of these complications can be attributed to the previous therapy patients have received, many are due to the unique aspects of HSCT, including conditioning regimen, use of total body irradiation (TBI), and the presence of graft versus host disease (GVHD).

For those patients identified as low risk through pre-HSCT MRD analysis, there may be a role for reduced intensity conditioning. Prior to the routine use of MRD, a retrospective analysis of pediatric patients with ALL who received TBI as part of their conditioning regimen demonstrated an improved overall and leukemia-free survival, compared to those who did not receive irradiation [60]. Since this time, TBI has become standard in most conditioning regimens for ALL. Given the long-term complications associated with TBI that are unique to growing and developing pediatric patients, there is much interest in reducing the radiation dose or eliminating this modality from the conditioning regimen altogether. 

Even more so, we know that there is a subgroup of children who relapse late (greater than 36 months following diagnosis) that can be cured with conventional chemotherapy [61]. Using MRD, we could potentially identify additional subgroups of patients that may achieve a cure without HSCT. Patients of specific interest for treatment reduction would be those with no MRD detected pre-HSCT by HTS-PCR, as this is our most sensitive modality to date. The absent MRD may represent residual disease that is below our level of detection but incapable of re-propagating leukemia or, alternatively, may represent complete clearance of malignant cells from the body. Support for this concept is given by the study from Pulsipher et al., previously discussed, that showed 0 of 22 patients with negative MRD using HTS-PCR prior to transplant went on to relapse, whereas 9 of the 19 patients with positive MRD prior to transplant went on to relapse [49].

### 4.2. Approaches to Eliminate Pre- and Post-HSCT MRD

Based on the evidence reviewed above, the presence of MRD pre-HSCT (at any level) increases the risk of relapse. Thus, this prognostic finding invites the opportunity to identify new strategies to eliminate pre-HSCT MRD, aiming to improve post-HSCT outcomes. At the most basic level, one would hypothesize that by eradicating pre-HSCT MRD we should be able to reduce the risk of relapse in a given patient. This idea is supported by a study from Balduzi et al. that showed a survival benefit in those pediatric patients with a positive pre-HSCT MRD who received further intensified chemotherapy [47]. They identified a small cohort of 13 patients with positive pre-HSCT MRD who, based on lack of significant co-morbidities, qualified for 1–2 blocks of fludarabine, cytarabine, and a liposomal anthracycline. Of these 13 patients, eight were able to achieve an MRD <10^−4^ prior to HSCT. Ten of the 13 patients were able to remain in long-term remission, with all three of the relapses occurring in the patients unable to achieve a negative MRD prior to HSCT. In addition, a small case series of eight pediatric patients reported improved outcomes in patients with ALL and pre-HSCT MRD who received bridging therapy prior to HSCT, with low dose clofarabine, cyclophosphamide, and etoposide [62]. All eight patients had a reduction in MRD detected via MFC with 6/8 becoming MRD negative (MRD < 0.01%) after a single cycle of bridging therapy, reporting a 1-year OS of 62.5%. These combined data would support the value of eliminating pre-HSCT MRD to improve outcomes. However, we must be cautious in giving more chemotherapy to patients prior to HSCT based on the presence of MRD, as we increase their risk for treatment-related complications, which may ultimately prevent them from receiving HSCT or impact post-HSCT complications, including TRM.

Identifying patients at high risk of relapse, based on their pre-HSCT MRD status, can allow us to prepare for the post-HSCT time period. Alternatively, by following post-HSCT MRD serially we can react to an impending relapse. Traditional salvage therapy for relapse post-HSCT has generally focused on more intensive chemotherapy and/or a second HSCT. However, a large percentage of patients post-HSCT are unable to tolerate such an intensive approach. In addition, this may be further compounded by the presence of GVHD. Therefore, a more common approach is to optimize the immunotherapeutic benefits of a GVL effect.

Given the multitude of challenges managing relapse ALL post-HSCT, there is growing interest in targeting pre-HSCT MRD with novel therapeutics. This approach is called bridging therapy. The goal of bridging therapy is to provide reasonably well tolerated therapy targeted toward the residual disease, with hopes of significantly reducing or eliminating it. Additionally, such agents may have a similar role in the post-HSCT period for patients defined as high-risk, based on their peri-HSCT MRD. Examples of bridging therapies include nucleoside analogues, epigenetic modifiers, molecularly targeted agents, and immune based therapies. The use of TKIs in patients with Ph+ ALL and Ph-like ALL is a specific example of current attempts to utilize a molecularly-targeted therapy to eliminate MRD during the peri-HSCT setting [63]. Therapies of particular interest are immune-based therapies that have the potential to enhance the GVL effect. A specific example is the bi-specfic T-cell engager, blinatumomab, which redirects T cells for tumor killing by targeting CD3 on the T cells and CD19 on leukemia cells. In a phase I/II study in pediatric patients with relapsed/refractory ALL, 14/70 (20%) patients achieved negative MRD within the first two cycles of the recommended blinatumomab dose [64]. A COG phase III randomized trial is currently exploring the efficacy of blinatumomab during the peri-HSCT setting for patients with relapsed ALL (NCT02101853). Another example of an immune-based therapy, which is being investigated in a similar fashion, is chimeric antigen receptor T-cell therapy (CART) (e.g., CTL019). These former two strategies work via the adaptive immune system and could therefore only be utilized post-HSCT once immunosuppression has been tapered off, or given pre-HSCT. Conversely, examples of an immune-based therapy that does not necessarily rely on an intact immune system are the monoclonal antibodies, which are designed to target tumor associated antigens (e.g., moxetumomab pasudotox and inotuzumab ozogamicin). However, such agents will need to be tested during the peri-HSCT period in larger trials to determine their toxicity, efficacy, and whether they will have a role in managing pediatric patients with ALL and pre- or post-HSCT MRD. 

### 4.3. Immune Suppression Modulation

It has long been known that a major benefit of allogeneic HSCT comes from the potential for a GVL effect [65]. GVL is mediated by donor T- and NK-cells that naturally target host antigens, similar to the graft-versus-host effect. The role of GVL seems to play a greater role in myeloid leukemias, such as chronic myeloid leukemia (CML) and AML, but its role in pediatric ALL has also been well documented [65,66,67]. The benefit of this effect must be balanced with the risk of lethal GVHD, and this balance comes in the way of donor selection and immune suppression. 

The role of the GVL effect is probably best evidenced by studies that show those patients who experience GVHD have a lower risk of relapse [65,67,68,69,70]. The impact of GVHD on relapse has been further emphasized in several of the studies following peri-HSCT MRD. In particular, Pulsipher et al. showed, in the COG ASCT0431 trial, that among the 19 patients with positive MRD pre-HSCT, there was a 73% probability of a 2-year relapse in those patients who didn’t experience acute GVHD by day 55 post-HSCT, versus a 17% probability in those who did experience acute GVHD of any grade (*p* = 0.02) [49].

It is, therefore, appealing to attempt to harness the GVL effect in the setting of MRD through the modulation of post-HSCT immunosuppressive therapy. If a patient is going to experience GVHD, they will typically do so in the first two months following HSCT [71]. Therefore, for those patients identified as high-risk for relapse based on pre-HSCT MRD, a lower dose of immunosuppression or planned rapid withdrawal of post-HSCT immunosuppression may lead to improved survival [72]. Similarly, for those patients who are found to be MRD-positive early in the post-HSCT setting and have not yet experienced GVHD, a rapid withdrawal of immune suppression may work against increasing MRD and re-achieve remission. This approach would permissively allow acute GVHD with the goal of unleashing the GVL effect. Several small studies have suggested a benefit to immune suppression modulation [47,50,53]. The benefit-to-risk ratio may be further enhanced with the advent of different graft manipulation techniques, which reduce the risk of GVHD while preserving the GVL effect, specifically T-cell depletion [73].

### 4.4. Donor Leukocyte Infusion

The tapering off of immune suppression post-HSCT will allow for the utilization of donor leukocyte infusions (DLI), as well as the use of certain novel therapeutics that are reliant on an intact immune system. The benefit of immune suppression withdrawal can be coupled to DLI for synergy [74,75]. The efficacy of DLI following relapse of ALL is generally inferior to the response seen in myeloid leukemias, but does still carry an anti-leukemia effect [76,77,78,79,80,81]. This limited utility appears more significant in B-ALL than T-ALL and is likely the result of multiple factors, including the slow onset of therapeutic action from DLI, the high proliferative capacity of ALL, and the decreased immunogenicity that may be associated with lymphoblasts, compared to myeloblasts [82]. It is therefore plausible that the use of DLI during a period of low leukemic burden, specifically low MRD, may be more effective. Additionally, this may allow for lower T-cell doses, which may reduce the potential complications associated with DLI, which include marrow aplasia or GVHD, which can develop in >60% of patients who receive DLI [83].

The concept of DLI, based on the presence of MRD, was supported by a recent study by Rettinger et al., which utilized post-HSCT MRD and chimerism monitoring to guide pre-emptive immunotherapy [84]. Immunotherapy included discontinuation or tapering of immunosuppression in the early post-transplantation period (or DLI for those patients off immunosuppression). Candidates for immunotherapy included those with detectable MRD by PCR (>10^4^) or mixed chimerism (1% recipient signals in consecutive samples or >1% of recipient signals in a single sample). Twenty-three of the 89 (26%) patients received immunotherapy based on chimerism and MRD analysis (*n* = 19), or MRD analysis alone (*n* = 4). The 3-year EFS for these patients was comparable to the 66 patients who did not receive immune intervention, due to full donor chimerism and MRD negativity (0.69 vs. 0.69). Additionally, none of the four patients who received immune intervention based solely on detectable MRD went on to relapse or die from treatment-related mortality. There was no increased incidence of acute GVHD in the treatment group, which was attributed to a lower initial T-cell dose in the DLI. This study not only may serve as a proof of principle, utilizing MRD to guide pre-emptive intervention, but also supports the concept of utilizing lower cell doses to reduce the risk of acute GVHD in this patient group.

In contrast, Lankester et al. performed a similar prospective trial, utilizing pre-HSCT MRD to identify patients for post-HSCT immune intervention. Using an MRD level of ≥10^−4^, they identified 18 children who received early cyclosporine A tapering followed by DLI. While this intervention may have delayed the timing of the relapse, the group reported that the EFS did not differ from that of historic controls who received no intervention [85]. Differences between these two studies can be attributed to a number of factors, including small numbers, heterogeneity of patients, timing of MRD analysis, and utilization and frequency of chimerism analysis. This discrepancy highlights the difficulty of assessing interventions in this diverse patient population.

## 5. Conclusions

Studies through numerous cooperative groups, as well as single institutions (including heterogeneous patient populations and different MRD modalities) have all consistently supported the role of conventional MRD assessment in the peri-HSCT setting as prognostic of relapse for pediatric patients with ALL. This includes the recent evidence that HTS-PCR, with its greater level of sensitivity, may further increase this prognostic significance, due to its ability to identify patients who otherwise would be identified as MRD-negative with conventional techniques. While patients with the presence of peri-HSCT MRD fair worse than their MRD negative counterparts, they do have improved outcomes compared to those with frank disease (>5% blasts). Therefore, the presence of MRD should not necessarily preclude transplant but rather could be used as a tool to guide peri-HSCT intervention. With growing evidence to support utilization of peri-HSCT MRD to guide further therapeutic intervention, focused primarily on harnessing the GVL effect, MRD assessment will soon become routine both pre and post-HSCT. The ideal way to identify the best therapeutic approach to manage peri-HSCT MRD in pediatric ALL would be through randomized clinical trials to identify whether pre-HSCT bridging therapies, post-HSCT interventions, or a combination of both provides the best outcomes in this high-risk group. As typically is the issue with this disease population, we are constrained by small numbers of patients and diverse demographics, making large prospective studies in patients with peri-HSCT MRD difficult. 

In summary, MRD remains the single most prognostic risk factor in B-ALL, which includes MRD identified either pre- or post-HSCT, and identifying methods to improve outcomes for these patients is urgently needed.

## Figures and Tables

**Table 1:** Modalities used to assess MRD.

Modality	Target	Sensitivity	Turnaround	Standardization	Diagnostic Sample	Patient-Specific Assays
**MFC**	Aberrant immunophenotype	10^−4^	Fast	Non-standardized (unless on study)	No	No
**ASO-PCR**	Ig/TCR variable junctional region	10^−4^–10^−5^	Slow	Standardized	Yes	Yes
**HTS-PCR**	Ig/TCR V-D-J gene	10^−6^	Medium	Non-standardized	Ideally	No

Note: MFC, multicolor flow cytometry; ASO-PCR, Allele-Specific Oligonucleotide Polymerase Chain Reaction; HTS-PCR, High Throughput Sequencing Polymerase Chain Reaction.

**Table 2:** Studies supporting the prognostic significance of MRD prior to HSCT.

Author	Year	Study Type	Technique	Sensitivity	*N*	Age, Years, Median (Range)	Remission	Results
**Knechtli [32]**	1998	R	PCR	<10^−3^–10^−5^	64	<18	CR1, CR2	2-year EFS 73% MRD− vs. 0% MRD+ *p* < 0.001
**Van der Velden [33]**	2001	R	PCR	<10^−4^	17	<15	CR1, CR2	5-year RFS 80% MRD− vs. 33% MRD+
**Sanchez [34]**	2002	P	MCF	<10^−4^	24	18 (3–49)	≥CR1	2-year RFS 73% MRD− vs. 33% MRD+ *p* = 0.03
**Bader [35]**	2002	R	PCR	<10^−4^	41	9.8 (1.5–17.8)	≥CR1	5-year EFS 78% MRD− vs. 32% MRD+ *p* = 0.011
**Krejci [36]**	2003	R	PCR	<10^−4^	140	<19	≥CR1	5-year EFS 75.2% MRD− vs. 29.8% MRD+
**Imashuku [37]**	2003	P	PCR	<10^−4^	95	9 (0.3–20)	Not remission, ≥CR1	Available data in 19 relapses, all 19 were MRD+
**Goulden [38]**	2003	R	PCR	<10^−4^	64	Pediatric	≥CR1	3-year EFS 73% MRD− vs. 17% MRD+ *p* < 0.001
**Sramkova [39]**	2007	P	PCR	<10^−4^	25	1.1–19	Partial remission, CR1, CR2	EFS 94% MRD− vs. 13% MRD+ *p* < 0.001
**Paganin [40]**	2008	P	PCR	<10^−4^	60	5 (0.6–17)	CR2	3-year EFS 73% MRD− vs. 19% MRD+ *p* < 0.05
**Bader [41]**	2009	P	PCR	10^−4^	91	11.1 (3–22.6)	CR2, CR3	3-year EFS 60% MRD− vs. 27% MRD+
**Elorza [42]**	2010	P	MCF	10^−4^	31	7 (<1–16)	≥CR1	2-year EFS 74% MRD− vs. 20% MRD+
**Leung [43]**	2012	R	MFC	10^−4^	64	11.3 (0.6–25.1)	≥CR1	5-year OS 87.5% MRD− vs. 48.5% MRD+
**Ruggeri [44]**	2012	R	PCR/MFC	10^−3−5^	170	6.5 (<1–17)	CR1,CR2, CR3	4-year CIR 24% MRD− vs. 39% MRD+
**Bachanova [45]**	2012	P	MFC	10^−3^	86	20 (6–63)	CR1, CR2, CR3	2-year RR 26% MRD− vs. 30% MRD+
**Shah [46]**	2014	R	MFC	10^−4^	34	<21	CR2	RR 35% MRD− vs. 64% MRD+
**Balduzzi [47]**	2014	P	PCR	10^−4^	82	8 (<1–20)	CR1, CR2, CR3	5-year EFS 77.7% MRD− vs. 30.8% MRD+ *p* < 0.001
**Bar [48]**	2014	R	MCF	10^−3^–10^−4^	153 (62 ped)	24.6 (0.6–61.8)	≥CR1	3-year EOR 17% MRD− vs. 38% MRD+
**Pulsipher [49]**	2015	R	HTS-PCR	10^−6^	41	1–21	CR1, CR2	2-year RR 0% MRD− vs. 53% MRD+
**Sutton [50]**	2015	P	PCR	10^−4^	69	Pediatric	CR1, CR2, CR3	LFS 83% MRD− vs. 41% MRD+
**Eckert [51]**	2015	P	PCR	10^−3^	71	Pediatric	CR1	DFS 58% MRD− vs. 26% MRD+

Note: R, Retrospective; P, Prospective; PCR, Polymerase Chain Reaction; MCF, Multicolor Flow Cytometry; HTS, High Throughput Sequencing; EFS, Event Free Survival; RFS, Relapse Free Survival; OS, Overall Survival; CIR, Cumulative Incidence of Relapse; RR, Relapse Rate; EOR, Estimates of Relapse; LFS, Leukemia Free Survival; DFS, Disease Free Survival.

**Table 3:** Studies supporting the prognostic significance of post-HSCT MRD.

Author	Year	Study Type	Technique	Sensitivity	*N*	Age, Years, Median (Range)	Remission	Results
**Sanchez [34]**	2002	P	MCF	<10^−4^	40	18 (3–49)	≥CR1	RR3% MRD− vs. 88% MRD+
**Imashuku [37]**	2003	P	PCR	<10^−4^	95	9 (0.3–20)	Active disease, ≥CR1	RR 26% MRD− vs. 27% MRD+ (*p* = 1)
**Zhao [52]**	2012	P	MFC	<10^−4^	139 (35 ped)	24 (4–55)	≥CR1	EFS 80% MRD− vs. 54% MRD+ (*p* = 0.001)
**Balduzzi [47]**	2014	P	PCR	10^−4^	82	8 (<1–20)	CR1, CR2, CR3	5-yr EFS 40.3% MRD+
**Bar [48]**	2014	R	MCF	10^−3^–10^−4^	144	24.6 (0.6–61.8)	≥CR1	HR 7.47 risk of relapse if MRD+
**Bader [53]**	2015	P	PCR	10^−4^	113	Pediatric	No remission and ≥CR2	MRD inversely correlated with EFS at all time points (*p* = 0.004)
**Pulsipher [49]**	2015	R	HTS	10^−6^	53	1–21	CR1, CR2	RR 13% MRD− vs. 73% MRD+
**Sutton [50]**	2015	P	PCR	10^−4^	47	Pediatric	CR1, CR2, CR3	LFS 67% MRD− vs. 36% MRD+

Note: R, Retrospective; P, Prospective; PCR, Polymerase Chain Reaction; MCF, Multicolor Flow Cytometry; HTS, High Throughput Sequencing; RR, Relapse Rate; EFS, Event Free Survival; HR, Hazard Ratio; LFS, Leukemia Free Survival.
